# Serum Prohepcidin Levels Are Lower in Patients with Atrophic Gastritis

**DOI:** 10.1155/2013/201810

**Published:** 2013-02-28

**Authors:** Hyung-Keun Kim, Eun-Chul Jang, Ju-Ok Yeom, Sun-Young Kim, Hyunjung Cho, Sung Soo Kim, Hiun-Suk Chae, Young-Seok Cho

**Affiliations:** ^1^Department of Internal Medicine, Uijeongbu St. Mary's Hospital, The Catholic University of Korea College of Medicine, Uijeongbu 480717, Republic of Korea; ^2^MOT Cluster, Korea University of Technology and Education, Cheonan 330708, Republic of Korea

## Abstract

*Background/Aim*. Hepcidin, an iron regulatory hormone, is increased in response to inflammation and some infections. We investigated the relationships among serum prohepcidin, iron status, *Helicobacter pylori* infection status, and the presence of gastric mucosal atrophy. *Methods*. Seventy subjects undergoing esophagogastroduodenoscopy underwent multiple gastric biopsies, and the possibility of *H. pylori* infection and the degree of endoscopic and histologic gastritis were investigated. In all subjects, serum prohepcidin and iron parameters were evaluated. *Results*. No correlations were observed between serum prohepcidin levels and the other markers of anemia, such as hemoglobin, serum iron, ferritin, and total iron binding capacity. Serum prohepcidin levels were not significantly different between the *H. pylori*-positive group and the *H. pylori*-negative group. Serum prohepcidin levels in atrophic gastritis patients were significantly lower than those in subjects without atrophic gastritis irrespective of *H. pylori* infection. *Conclusion*. Serum prohepcidin levels were not altered by *H. pylori* infection. Serum prohepcidin levels decrease in patients with atrophic gastritis, irrespective of *H. pylori* infection. It suggests that hepcidin may decrease due to gastric atrophy, a condition that causes a loss of hepcidin-producing parietal cells. Further investigations with a larger number of patients are necessary to substantiate this point.

## 1. Introduction

Human hepcidin, a 25-amino-acid peptide first identified in human urine and plasma that is secreted mainly from the liver, exerts *in vitro* antibacterial and antifungal activities [1, 2]. Prohepcidin, an 84-amino-acid precursor form of hepcidin, is found in blood [3]. Hepcidin is an acute-phase reactant, and its expression is upregulated via interleukin (IL)-6 during bacterial infection and inflammation [4]. In addition, hepcidin plays a major role in homeostatic regulation of iron metabolism. This peptide acts by binding to the cellular iron exporter ferroportin and inducing its internalization and degradation, thus trapping iron in enterocytes, macrophages, and hepatocytes [5]. Hepcidin synthesis is increased by iron overloading and decreased by iron deficiency [6, 7]. 

*Helicobacter pylori* infection with or without coexisting autoimmune gastritis has been implicated in several recent studies as an important cause of iron deficiency anemia (IDA) in patients with unexplained IDA [8]. The possible pathogenic mechanisms include occult blood loss secondary to chronic erosive gastritis, decreased iron absorption secondary to atrophy-associated gastric hypochlorhydria, and increased iron uptake and utilization by *H. pylori* [9]. Moreover, iron-deficient patients who have *H. pylori* infection seem not to respond well to oral iron therapy until the bacterium had been eradicated [10–12]. This hypothesis was confirmed by a study showing impaired absorption of iron after oral loading in infected subjects and reversion to normal after eradication [13]. It has been suggested that the reason for the failure of patients with *H. pylori* infection to respond to iron might be the production of hepcidin or hepcidin mimetics by microorganisms [14, 15]. A recent study showed that gastric hepcidin expression was significantly upregulated in *H. pylori*-infected patients and normalized by *H. pylori* eradication [16]. The study also demonstrated that gastric hepcidin was localized in parietal cells, which regulate gastric acid production. 

Serum prohepcidin concentrations are significantly decreased in patients with hereditary hemochromatosis [3], increased with declining kidney function [17], and are positively correlated with hematocrit in chronic hemodialysis patients [18]. In this study, we evaluated the relationships among serum prohepcidin, iron status, *H. pylori* infection status, and the presence of gastric mucosal atrophy.

## 2. Materials and Methods

### 2.1. Study Population

This was a single center, observational case-control study including 70 subjects who underwent routine endoscopic examination of gastrointestinal symptoms at the Uijeongbu St. Mary's Hospital between September 2005 and August 2006. Exclusion criteria were previous eradication therapy or the use of bisthmus compounds, proton pump inhibitors, antibiotics, or antisecretory drugs within the previous 2 months. Additional exclusion criteria were pregnancy or lactation, severe systemic illness, manifest clotting disorders or the use of anticoagulants, and a history of blood transfusion or iron supplement therapy. 

### 2.2. Diagnosis of *H. pylori* Infection

During endoscopy, four biopsies (two from the antrum, two from the corpus) were taken. Hematoxylin and eosin (HE) staining and Giemsa staining were performed using serial sections of four specimens. The sections were independently assessed by two blinded pathologists. The ^13^C-Urea Breath Test (UBT) was performed after an overnight fast or at least an 8 h fast. A baseline breath sample was placed into a collection tube. An aliquot of 75 mg of ^13^C-urea dissolved in 75 mL of citric acid solution was given orally (Helikit; Isodiagnostika, Edmonton, Canada). Another breath sample was collected after 30 min. Breath samples were subsequently analyzed to determine the ^13^C/^12^C ratio by mass spectrometry (HeliView; MediChems, Seoul, Republic of Korea). The ^13^C/^12^C ratio of each breath sample was expressed as a milli-percentage (‰). Change in the ^13^C value over baseline was expressed as delta ^13^C. A positive result was defined as an increase of >4‰. Patients were considered to be negative for *H. pylori* if both histological examination and ^13^C-UBT results were negative. Patients were considered to be positive for *H. pylori* if any one of the tests was positive. 

### 2.3. Diagnosis of Atrophic Gastritis

Atrophic changes of the gastric mucosa on endoscopy were graded according to Kimura-Takemoto classification [19]. Atrophic patterns were classified into eight types by the location of the atrophic border. The C-0 pattern shows an endoscopically normal stomach without atrophic change in any area. C-1, -2, and -3 denote closed-type atrophic patterns. In the C-1 type, atrophic changes are limited to the antrum. Atrophic borders lying on the lesser curvature of the lower body define the C-2 pattern, and those on the upper body define the C-3 pattern. Meanwhile, O-1, -2, and -3 denote open-type atrophic patterns. In the O-1 type, the atrophic border is located within the lesser curvature of the body; in the O-2 type, the border is located in the anterior and posterior walls; and in the O-3 type, the border is located in the greater curvature. A histological diagnosis of atrophic gastritis was made according to the updated Sydney System using a biopsy specimen taken from the lesser curvature of the lower body [20]. Gastric mucosal inflammation (mononuclear cell infiltration), inflammatory activity (neutrophil infiltration), atrophy, and intestinal metaplasia were each assessed semiquantitatively and graded as 0, 1, 2, or 3.

### 2.4. Laboratory Analysis

Blood samples were collected from all participants who had fasted overnight. Laboratory tests, including a complete blood count, total protein, albumin, hepatic and renal function tests, serum iron, total iron biding capacity (TIBC), and ferritin were performed using standard laboratory methods. Patients with a hemoglobin (Hb) of <14.0 g/dL (men) or <12.0 g/dL (women) were considered to be anemic, and ferritin concentrations of <30 *μ*g/L (men) or <13 *μ*g/L (women) were considered indicative of iron deficiency.

 The serum prohepcidin level was measured using the DRG Diagnostics Hepcidin Prohormone enzyme-linked immunosorbent assay (ELISA) (DRG Instruments GmbH, Marburg, Germany), according to the manufacturer's instructions. The employed antibody detects both the proregion and prohepcidin (aa 25–84). The sensitivity of the assay was 3.95 ng/mL, intra-assay coefficient of variation (CV) was 4.69%, and inter-assay CV was 4.82%.

### 2.5. Statistical Analysis

Values are expressed as mean ± standard deviation (SD). Continuous data were compared using an independent-samples *t*-test, whereas the categorical data were analyzed using *χ* ^2^ or Fisher's exact tests. For correlation analysis, the Spearman nonparametric correlation was used. Data were processed and analyzed with SPSS, version 12.0 (Chicago, IL, USA). A value of *P* < 0.05 was considered to indicate statistical significance for all tests.

### 2.6. Ethics Statement

This study protocol was approved by the Institutional Research Ethics Board of Uijeongbu St. Mary's Hospital of the Catholic University of Korea (IRB No. UCMC06BR006) and adhered to the Declaration of Helsinki. All of the study subjects completed an informed consent form before participating in the study. The informed consent was confirmed by the board.

## 3. Results

A total of 70 patients with gastrointestinal symptoms (29 men, 31 women; mean age, 41.7 years; range, 21–77 years) were included in the study. When the patients were separated into *H. pylori*-positive (*n* = 35) and *H. pylori*-negative (*n* = 35) groups, no significant differences in the hematological or biochemical parameters were observed between the two groups. However, a difference in atrophic gastritis was identified, which was significantly more frequent (*P* < 0.001) in the *H. pylori*-positive group than in *H. pylori*-negative group (71.4% versus 28.6%, resp.). Anemia was diagnosed in nine (12.9%) patients, three of whom were *H. pylori*-positive and six of whom were *H. pylori*-negative. Among anemic patients, only three in the *H. pylori*-negative group had iron deficiency. The mean serum prohepcidin level was 241.4 ng/mL (SD, 58.6; range, 137–401) among all subjects, 238.8 ng/mL (SD, 60.9; range, 144–401) in the *H. pylori*-positive group, and 244.1 ng/mL (SD, 57.0; range, 137–359) in the *H. pylori*-negative group. The serum prohepcidin level correlated with the hemoglobin (*r* = 0.355, *P* = 0.036) and ferritin (*r* = 0.371, *P* = 0.028) levels in the *H. pylori*-positive group. No correlation was observed between the serum prohepcidin level and the other markers of anemia, such as hemoglobin, serum iron, ferritin, and TIBC, in the *H. pylori*-negative group.

**Figure 1:**
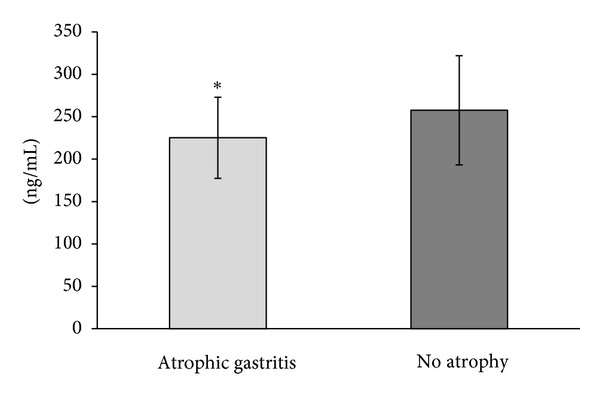
Serum prohepcidin levels in 35 atrophic gastritis patients and in 35 subjects without atrophic gastritis.

 Twenty-five *H. pylori*-negative subjects showed an endoscopically normal stomach with a C-0 atrophic border and histologically normal fundic mucosa, and 10 subjects showed mild atrophic change (C-1). Ten *H. pylori*-positive subjects showed no atrophic changes and histologically normal fundic mucosa, and 25 subjects showed various endoscopic atrophic changes (C-1, *n* = 14; C-2, *n* = 4; and C-3, *n* = 7). The descriptive statistics are reported in Table 1. Patients with atrophic gastritis were older than subjects without atrophic gastritis. However, there was no correlation between the age and the serum prohepcidin level among all subjects. Serum prohepcidin levels were significantly lower in atrophic gastritis patients than in subjects without atrophic gastritis (225.2 ± 47.8 versus 257.6 ± 64.4 ng/mL, *P* = 0.020) irrespective of *H. pylori* infection (Figure 1). However, in atrophic gastritis patients, the degree of atrophy was not correlated to the serum prohepcidin level (*P* = 0.058). No correlation was observed between the serum prohepcidin level and the other markers of anemia, such as hemoglobin, serum iron, ferritin, and TIBC, in patients with and without atrophic gastritis (Table 2). 

## 4. Discussion

Since its discovery, hepcidin has attracted the attention of investigators because of its ability to regulate iron metabolism and exert antimicrobial activity against numerous bacteria and fungi [1, 2, 4–7]. In the present study, the serum level of prohepcidin, a hepcidin precursor, and its relationship with iron metabolism and *H. pylori* infection were evaluated. There was no relationship between prohepcidin, iron deficiency parameters, and *H. pylori* infection. 

 Hepcidin plays a major role in the iron regulatory mechanism through inhibition of iron export from enterocytes, macrophages, and hepatocytes [5]. Hepcidin levels increase in response to iron loading, reducing intestinal iron absorption and inhibiting iron release from stores [21]. Meanwhile, iron deficiency produces low hepcidin levels, resulting in enhanced iron absorption and iron mobilization from stores. In addition, hepcidin is induced by inflammation, causing its sequestration in stores [7]. The resulting iron decrease contributes to anemia in chronic disease. The relationship of hepcidin to disorders of iron metabolism has been established via the measurement of urinary hepcidin concentrations using immune-dot [7], sodium dodecyl sulfate polyacrylamide gel electrophoresis (SDS-PAGE), western blot [4], and surface-enhanced laser desorption/ionization time-of-flight mass spectrometry (SELDI-TOF-MS) [22]. Recent studies reported two types of hepcidin assays for the semiquantitative or quantitative determination in human serum. First, mass spectrometric assays detect the characteristic mass of the active 25-amino-acid hepcidin species or its fragments [23–25]. However, these assays require access to specialized equipment and are not widely available. Second, an ELISA specific for the refolded, mature 25-amino-acid form was developed [26]. The serum hepcidin level by ELISA was inversely correlated with iron absorption from supplemental and food-based nonheme iron sources in iron-replete healthy women [27]. However, subsequent evaluation is required to prove the usefulness of this method. 

 In the present study, we measured serum prohepcidin levels; this is one of the limitations of this study. Prohepcidin is far more immunogenic than hepcidin, and a prohepcidin ELISA is commercially available. Our study failed to show any association between serum prohepcidin concentrations and iron deficiency parameters. These results are in agreement with those of previous studies in which the serum prohepcidin concentration was correlated poorly with markers of iron homeostasis, such as intestinal iron absorption [27–29]. In addition, a relationship between serum concentrations of prohepcidin and those of hepcidin 25 was not found [27]. However, another study reported a significant positive correlation between prohepcidin and hepcidin serum levels [30]. 

 An association between *H. pylori* infection and IDA has been reported [8–12]. Our study showed that anemia was not increased in subjects with *H. pylori* infection and showed no relationship with hepcidin. This may be due to the small sample size, the fact that the study cohort was fairly uniform, and the fact that most subjects had normal iron stores. Nevertheless, our data do not support the proposal that hepcidin plays a key role in the primary mechanism of *H. pylori*-induced anemia. Previous studies have demonstrated that hepcidin and prohepcidin serum levels by ELISA were not altered by *H. pylori* infection or eradication even when hepcidin was detected in human gastric juice [16, 31, 32]. These findings suggest that hepcidin may exert local rather than systemic functions.

Schwarz et al. recently reported a new role for hepcidin in the stomach [16]. In this study, quantitative RT-PCR demonstrated abundant hepcidin expression in the fundus/corpus part of the glandular stomach in mice, rats, and humans. Hepcidin was localized in gastric parietal cells by immunofluorescence staining and *in situ* hybridization. Gastric hepcidin expression in patients and in AGS cells was significantly upregulated during *H. pylori* infection. In addition, *H. pylori* eradication resulted in normalization of hepcidin expression levels. Moreover, hepcidin-knockout mice displayed decreased H^+^/K^+^-ATPase gene expression, significant bacterial overgrowth, and reduced gastric gene expression. These findings suggest that hepcidin regulates gastric acid production and may contribute to the development of gastric ulcers. In the present study, atrophic gastritis was found to be present in 50%. In Korea, the seroprevalence of *H. pylori* was high (59.6%) in the Korean population among asymptomatic Korean adults in 2005 [33]. In addition, the prevalence of atrophic gastritis in the antrum and body was 42.5% and 20.1%, respectively, in Korean population without significant gastroduodenal disease [34]. We found that serum prohepcidin levels decreased in subjects with gastric atrophy, irrespective of *H. pylori* infection. This finding might be explained by the loss of hepcidin-producing cells caused by gastric atrophy. However, we did not find that serum prohepcidin levels are related to the degree of atrophy. In addition, correlation between the serum prohepcidin and the parameters for anemia was not found in patient with atrophic gastritis. This can be explained by the small number of patients enrolled and by the lack of evaluation of hepcidin expression in gastric tissues. A recent study demonstrated that gastric hepcidin expression decreased in hypergastrinemic ING-mice with chronic gastric *H. pylori* infection and resulted in the upregulation of the expression of various downstream iron absorption and efflux genes such as Ferroportin 1, Divalent metal transporter 1, and Transferrin receptor 1 [35]. These findings suggest that the decrease of gastric hepcidin expression due to the loss of hepcidin-producing parietal cells may function as an iron regulator. However, further studies regarding the functional role of hepcidin and iron transporter in the gastric mucosa are required because iron is mainly absorbed in the small intestine.

Pepsinogen, an aspartic proteinase secreted mainly by gastric cells, is classified immunologically as pepsinogen I (PG I) and pepsinogen II (PG II). Whereas PG I is secreted only from the gastric fundic mucosa, PG II is secreted from the cardiac, fundic, and antral mucosae of the stomach [36]. The effects of gastric atrophy on serum PG concentrations are lower PG I and stable or increased PG II levels, and this results in a lower PG I/II ratio [37]. Gastrin is another valid tool for detection of gastric body mucosal atrophy, and increased serum gastrin is a regular feature of atrophic body gastritis due to the loss of negative feedback by gastric acidity [38]. Further study is needed to evaluate the relationships between hepcidin and gastric mucosal atrophy using the gastrin and/or pepsinogen I/II ratio.

 In conclusion, our data suggest that serum prohepcidin levels were not altered by *H. pylori* infection. Serum prohepcidin levels decreased in patients with atrophic gastritis, irrespective of *H. pylori* infection. It suggests that hepcidin may decrease due to gastric atrophy, a condition that causes a loss of hepcidin-producing parietal cells. Further studies with a larger number of atrophic gastritis patients are necessary to better investigate the relationship between hepcidin and atrophic gastritis.

## Figures and Tables

**Table 1:** Demographic feature, hematological and biochemical parameters in the study groups.

	Atrophic gastritis-positive (*n* = 35)	Atrophic gastritis-negative (*n* = 35)	*P* value
Male gender	*n* = 17 (48.6%)	*n* = 12 (34.3%)	0.231
Age	45.9 ± 10.6	37.4 ± 11.9	0.002
*H. pylori* positivity	*n* = 25 (71.4%)	*n* = 10 (28.6%)	0.000
Hb, g/dL	14.0 ± 1.72	13.0 ± 2.55	0.062
Serum iron, *μ*g/dL	113.0 ± 42.49	91.9 ± 55.87	0.080
Serum ferritin, *μ*g/L	111.74 ± 136.58	83.24 ± 104.50	0.331
TIBC, *μ*mol/L	303.7 ± 40.56	331.46 ± 63.29	0.033
Serum prohepcidin, ng/mL	225.2 ± 47.84	257.6 ± 64.40	0.020

**Table 2:** Correlation between the serum prohepcidin and the parameters for anemia in patients with and without atrophic gastritis.

	Atrophic gastritis-positive (*n* = 35)		Atrophic gastritis-negative (*n* = 35)	
	Correlation coefficient (*r*)	*P*-value	Correlation coefficient (*r*)	*P*-value
Prohepcidin X Hb	0.036	0.837	0.267	0.120
Prohepcidin X SI	−0.200	0.250	0.026	0.880
Prohepcidin X SF	0.224	0.195	0.197	0.256
Prohepcidin X TIBC	0.171	0.327	0.104	0.552

*R*: Spearman coefficient; Hb: hemoglobin; SI: serum iron; SF: serum ferritin; TIBC: total iron binding capacity.
